# Segmentation of Pancreatic Subregions in Computed Tomography Images

**DOI:** 10.3390/jimaging8070195

**Published:** 2022-07-12

**Authors:** Sehrish Javed, Touseef Ahmad Qureshi, Zengtian Deng, Ashley Wachsman, Yaniv Raphael, Srinivas Gaddam, Yibin Xie, Stephen Jacob Pandol, Debiao Li

**Affiliations:** Cedars-Sinai Medical Center, Biomedical Imaging Research Institute, Los Angeles, CA 90048, USA; touseefahmad.qureshi@cshs.org (T.A.Q.); zengtian.deng@cshs.org (Z.D.); ashley.wachsman@cshs.org (A.W.); yaniv.raphael@cshs.org (Y.R.); srinivas.gaddam@cshs.org (S.G.); yibin.xie@cshs.org (Y.X.); stephen.pandol@cshs.org (S.J.P.)

**Keywords:** pancreatic subregions segmentation, pancreas segmentation, CT abdominal scans

## Abstract

The accurate segmentation of pancreatic subregions (head, body, and tail) in CT images provides an opportunity to examine the local morphological and textural changes in the pancreas. Quantifying such changes aids in understanding the spatial heterogeneity of the pancreas and assists in the diagnosis and treatment planning of pancreatic cancer. Manual outlining of pancreatic subregions is tedious, time-consuming, and prone to subjective inconsistency. This paper presents a multistage anatomy-guided framework for accurate and automatic 3D segmentation of pancreatic subregions in CT images. Using the delineated pancreas, two soft-label maps were estimated for subregional segmentation—one by training a fully supervised naïve Bayes model that considers the length and volumetric proportions of each subregional structure based on their anatomical arrangement, and the other by using the conventional deep learning U-Net architecture for 3D segmentation. The U-Net model then estimates the joint probability of the two maps and performs optimal segmentation of subregions. Model performance was assessed using three datasets of contrast-enhanced abdominal CT scans: one public NIH dataset of the healthy pancreas, and two datasets *D*_1_ and *D*_2_ (one for each of pre-cancerous and cancerous pancreas). The model demonstrated excellent performance during the multifold cross-validation using the NIH dataset, and external validation using *D*_1_ and *D*_2_. To the best of our knowledge, this is the first automated model for the segmentation of pancreatic subregions in CT images. A dataset consisting of reference anatomical labels for subregions in all images of the NIH dataset is also established.

## 1. Introduction

The pancreas adopts several morphological changes during the development of pancreatic cancer (PC) due to the underlying biological mutations [1,2,3,4]. Identifying such changes can efficiently assist in the early diagnosis, monitoring of disease progression, and treatment planning of PC. However, such alterations are subtle and unique to each pancreatic subregion, which includes the head (H), body (B), and tail (T), and thus require analysis at the regional level. For instance, tumor histology differs across pancreatic subregions [5,6] (e.g., H tumors are non-squamous, B/T tumors are squamous), causing spatial heterogeneity in the pancreas. This key difference causes several diversities, including tumor presentation (H: less aggressive, well-defined, B/T: more aggressive, poorly differentiated), associated symptoms (H: weight loss, B: upper abdominal pain, T: lower abdominal pain), drug response (H: more sensitive to Gemcitabine regimen, B/T: more sensitive to Fluorouracil regimen, and vice versa), and variable rates for metastasis (H: 42%, B: 68%, T: 84%), incidence (H: 71%, B: 13%, T: 16%), 2-year survival (H: 44%, B: 27%, T: 27%), and resection (H: 17%, B: 4%, T: 7%) [5,6,7,8,9,10].

Computed Tomography (CT) abdominal imaging is widely used for noninvasive examination of the morphology and texture of the whole pancreas and subregions [11] (the terms ‘pancreatic subregion’ and ‘subregion’ are used interchangeably). However, due to the small size, complex location, and the irregular anatomy of the pancreas, manual evaluation of CT scans does not fully appreciate valuable information and often leads to misdiagnosing PC [12,13]. Furthermore, subregional alterations are usually assessed as changes to the whole pancreas, which often results in statistical insignificance as the local microlevel changes mostly remain obscured when appraised globally. Additionally, manual outlining of subregions can be subjective, limiting the precise analysis of subregions.

The reliable and rigorous analysis of pancreatic morphology requires accurate and automated delineation of the pancreas and subregions, as manual segmentation is highly subjective, tedious, and prone to errors. Several automated methods [14,15,16] have been proposed for whole pancreas segmentation using CT images in recent years, obtaining satisfactory levels of accuracy. However, unfortunately, due to the lack of a clear need for subregional analysis, rigid criteria to specify subregional boundaries, and unavailability of ground truth labels, the subregional segmentation of pancreas in CT images has remained un-attempted. In the case of Magnetic Resonance images, there is only one technique [17], published recently, that attempted to segment pancreatic subregions using a delineated pancreas. Thus, the high application of CT abdominal imaging to PC diagnosis and management calls for automatic subregional segmentation in CT images to assist in a reliable subsequent assessment of subregions.

The key challenge for naïve segmentation models is the lack of distinct boundaries and edge information between any two adjacent subregions. Deep learning networks have been used for the zonal segmentation of several anatomical structures [18,19,20]. The performance of these techniques largely depends on the segmentation approach adopted. The 2D approaches usually have more slices to train the network and are less computationally expensive. However, for the current problem, the 2D approach would suffer challenges as the three subregions do not appear together in most of the 2D CT abdominal slices which limits the usefulness of that contextual information. On the contrary, 3D approaches consider the spatial and volumetric information of the substructure and are thus more stable and robust. However, 3D approaches require huge training data. Unfortunately, public CT datasets for the pancreas are rare and small, with no subregional labels. A more efficient approach is the systematic integration of anatomical and contextual information of the substructures to the base 3D model to perform a more enhanced and guided segmentation, even with a small dataset. Many models followed this approach and achieved desirable levels of performance, including [21].

This paper presents a fully automated method for the 3D segmentation of pancreatic subregions using outlined pancreas in CT images. A multistage anatomy-guided frame- work is proposed that generates a probability map for subregional segmentation using the Naïve Bayes model which makes use of length and volumetric proportions of subregions, based on their adjacency arrangement, followed by the incorporation of the probability map to the conventional 3D U-Net segmentation model [22] that performs enhanced segmentation. Model evaluation was performed on three datasets of contrast-enhanced abdominal CT scans, including the NIH public dataset [23] of the healthy pancreas, and two in-house datasets (one for each of the pre-cancerous and cancerous pancreas). Experimental results show a significant overlap between the predicted segmentation and reference labels with the mean overall Dice Sørensen Coefficient (*DSC*) reaching 94.5%, 95.6%, and 89.9% for the NIH, precancerous, and cancerous datasets, respectively. Compared to the results obtained using the U-Net model without integrating the map generated by the Bayes model, an average 8% *DSC* improvement is observed for all three sets, indicating the substantial gain achieved by incorporating the anatomical information. Also, a new dataset has been established that consist of subregional reference labels for the NIH image dataset.

To the best of our knowledge, this is the first model proposed for the automated segmentation of pancreatic subregions in CT images. The originality of the work consists in applying the anatomical information of subregional structures into the model. The reference labels dataset will be made publicly available or can be requested from authors.

## 2. Materials and Methods

### 2.1. Datasets

Model training and the validation of the proposed methodology were performed using the NIH dataset containing 82 contrast-enhanced abdominal CT scans, acquired from subjects with a healthy pancreas. Each scan has a resolution of 512 × 512 pixels on the x/y axis, while the number of sampling slices on the z-axis varies between 181 and 466, with the slice thickness varying between 0.5 and 1.0 mm. The dataset also comes with gold reference labels outlining the pancreas in all scans and has been used in many studies for the automatic segmentation of the pancreas.

Two additional in-house datasets (*D*_1_, *D*_2_) were obtained from the repository of the CSMC that consist of CT scans of same subjects with pre-cancerous and the cancerous pancreas respectively. Each dataset consists of 15 contrast-enhanced, multi-phase (predominantly portal venous phase), CT abdominal scans with identical x/y resolution to NIH scans and variable z-axis slices.

To generate reference labels for subregions in all scans of three datasets, two trained radiologists, with over 12 years of experience at the Department of Radiology, Cedars-Sinai Medical Center (CSMC), manually specified subregional boundaries. A three-step process was conducted to perform labeling and to ensure labeling consensus. First, both graders independently labelled subregions within the outlined boundary of pancreas in all scans. Approximately 84% labelling overlap was observed in both label sets. Second, both graders shared their labels with each other so the graders could review and update their original labels, resulting in 96% overall labelling consistency. Lastly, the remaining 4% of labels were finalized with mutual agreement of both graders.

### 2.2. Anatomy-Guided Subregional Segmentation

#### 2.2.1. Anatomy of the Pancreatic Subregions

Pancreatic subregions follow certain anatomical constraints that help specify the general spatial location of each subregion and the area it covers. Effectively utilizing the anatomical and geometrical dimensions of the pancreas can reduce segmentation errors.

**Figure 1:**
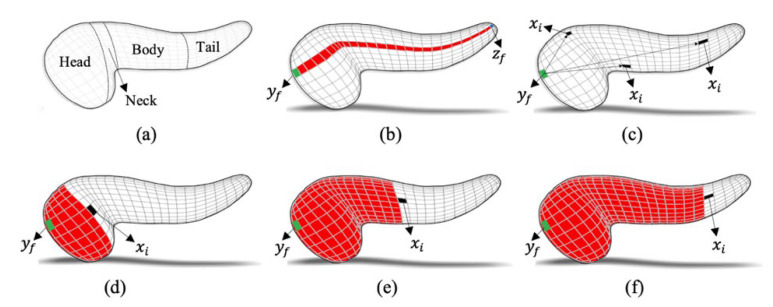
(**a**): Pancreas structure with head, neck, body, and tail region in a 3D mesh. (**b**): Length of the pancreas is shown as the shortest Euclidian distance (in red) between $y_{f}\;$(in green) and the pixel farthest from it (in blue). (**c**): three examples of pixel $x_{i}$ (in black). For each $x_{i}$, the α is the Euclidian distance between $y_{f}$ and $x_{i}$. (**d**–**f**): Examples of $x_{i}$ considered at three different locations. For each $x_{i}$, the β is the area (in red) between $y_{f}$ and $x_{i}$ in 3D space.

The pancreas is an accessory gland of the digestive system, situated across the posterior abdominal wall behind the stomach in the epigastric region, with both exocrine and endocrine functions [24]. In its complete form, it appears as a J-shaped (hockey stick-like) structure that is generally divided into three anatomical parts: head, body, and tail. The head is the expanded medial part that lies in the curve of the duodenum. The body continues and connects to a tapered tail, which is the last part of the pancreas. On the CT axial view, the head, body, and tail appear in the order of left to right. The pancreas anteroposterior diameter is observed as 1–3 cm, and length as 12–15 cm with head, body, and tail [25] covering 40%, 33%, 26% of its length, respectively. Figure 1a illustrates the anatomical structure of the three subregions.

#### 2.2.2. Bayesian Model for Soft Labels

Useful anatomical information assisting the segmentation process includes (a) the adjacency arrangement of the subregions, (b) the length proportion of each subregion to the whole pancreas and (c) the volumetric proportion of a subregion to the whole pancreas. The naïve Bayes model was trained to systematically integrate the latter two features (b, c), based on information from the former feature (a), and generate a probability map (soft label) for a CT abdominal image, specifying the likelihoods of each pancreas voxel to be associated to the head, body, and tail. Let $x_{i}$ represent a pixel in the pancreas region, then $P_{i}^{s}\left(s|x\right)$ is the probability for $x_{i}$ to be the part of the subregion s, where s is the subregional index (H, B, T), and i is the pixel index. The expression $P_{i}^{s}\left(s|x\right)$ is the joint probability and is calculated as $P_{i}^{s}\left(s|x\right)\propto P_{i}^{s}\left(s|\alpha \right)\cdot P_{i}^{s}(s|\beta )$.

The term $P_{i}^{s}\left(s|\alpha \right)$ is the conditional probability such that α (the length feature) is the pixel-wise shortest-possible Euclidian distance in the 3D pancreas region between pixels $x_{i}$ and $y_{f}$, whereas $y_{f}$ is the farthest pixel of the head from the pixels of the body and tail subregions. The feature α is expressed as percent proportion to avoid length variation of pancreas across different subjects and is given by α=α/l, whereas l is the total length of the pancreas and is the pixel-wise shortest-possible Euclidian distance in the 3D pancreas between $y_{f}$ and $z_{f}$ (the pixel farthest from $y_{f}$). Figure 1b provides the illustration. A high α value indicates that $x_{i}$ likely belongs to the tail, a moderate value suggests $x_{i}$ belongs to the body, whereas a small value shows that $x_{i}$ belongs to the head, illustrated in Figure 1c. Through a fully supervised learning process, the distribution parameters ($\mu_{\alpha }^{s},\;\sigma_{\alpha }^{s}$) for α obtained from pixels of all three subregions (s) in the training images, implies as $P_{i}^{s}\left(s|\alpha \right)$ = $P_{i}^{s}\left(\mu_{\alpha }^{s},\;\sigma_{\alpha }^{s}|s\right)\cdot P\left(s_{\alpha }\right)$. The quantity $P\left(s_{\alpha }\right)$ is the prior probability which is set to 0.33 to give an equal opportunity for $x_{i}$ to be associated to any subregion.

The term $P_{i}^{s}(s|\beta )$ is the conditional probability such that β (the volumetric feature) is the total number of pixels that lie between $x_{i}$ and $y_{f}$ on all three axes within the 3D pancreas region. The feature β expresses how far (area-wise) the $x_{i}$ is from $y_{f}\;$(the start of the head) in 3D space. A high β value indicates that $x_{i}$ is likely in the tail area, a moderate value suggests that $x_{i}$ is lying in the body area, whereas a small value shows that $x_{i}$ likely falls in the head area. Since the size of the pancreas differs across different subjects, the feature, β, is not used as an absolute value but expressed as a percentage to avoid the impact of variations and is calculated by β=β/A, whereas A is the total area (number of pixels) in the 3D pancreas region. Figure 1d–f provide the illustration. Through a fully supervised learning process, the distribution parameters ($\mu_{\beta }^{s},\;\sigma_{\beta }^{s}$) for β were obtained from pixels of all three subregions (s) in the training images. This implies to $P_{i}^{s}\left(s|\beta \right)$=$P_{i}^{s}\left(\mu_{\beta }^{s},\;\sigma_{\beta }^{s}|s\right)\cdot P\left(s_{\beta }\right)$. The prior probability $P\left(s_{\beta }\right)$ is set to 0.33 for any subregion.

#### 2.2.3. U-Net Model for Segmentation

The U-Net is based on commonly used region-based CNN (Convolutional Neural Network) for fast and precise segmentation of images, particularly when training data is limited or has a great deal of variability. The high efficiency and performance of the U-Net for the segmentation of several small and variable organs in medical images, including the pancreas, has been observed in previous studies [26].

In current work, the conventional 3D U-Net architecture [23] is used as a base model for the 3D segmentation of pancreatic subregions in CT images. The soft label obtained from the Bayes model is incorporated into the U-Net model for each corresponding image. The U-Net model estimates the joint probability map of two soft labels (generated by U-Net and Bayes) in each epoch iteration during training and finds the optimal segmentation based on the integrated probability map. Similarly for testing, the Bayes model generates soft labels for all testing images, and the U-Net incorporates these soft labels to U-Net-based soft labels, before performing the final segmentation.

## 3. Experimental Setup and Implementation

### 3.1. Data Preparation and Evaluation Criteria

All images in the three datasets were cropped down to remove non-pancreatic regions using the outlined pancreas in reference labels, resulting in a limited fixed image resolution of 218 × 239 × 288 in the x-, y-, and z-axes. The cropping did not remove any of the pancreas pixels in any image. All non-pancreas pixels within the cropped region were set to 0 intensity, whereas the intensities of pancreas pixels in each image were normalized to unity (i.e., 0–1) using linear scaling. No other preprocessing was undertaken on the data.

The NIH dataset was split into 4 roughly equally sized subsets, where 3 unique subsets (~60 scans) were used for model training, and the remaining ~20 scans for testing, in each of 4 folds. The *DSC* was calculated for performance evaluation in each fold. The *DSC* is a similarity metric between the prediction pixels set and the gold reference label set, with the mathematical form of *DSC* = 2*TP*/(2*TP* + *FP* + *FN*). For instance, to calculate the *DSC* for a subregion (e.g., head), all pixels from the head would be considered in the Positive (P) class, whereas all the pixels of the other two subregions (i.e., body, tail) would be considered from Negative (N) class.

### 3.2. Model Training

The Naïve Bayes probability model was implemented for a three-class problem in MATLAB (version 2021b). During each iteration of the training in four folds, the two features α and β from all the training ~60 scans were extracted for three subregions, and their distribution parameters were learned. Using the learned distribution parameters, a probability map $X_{j}^{n}$ (soft label) was generated for all training images, where j was the training image index and *n* was the training fold index. The soft label $X_{j}^{n}$ is a map indicating three-class likelihoods (normalized probabilities), one for each of three subregions, associated with each pancreas pixel i in the j image.

**Figure 2:**
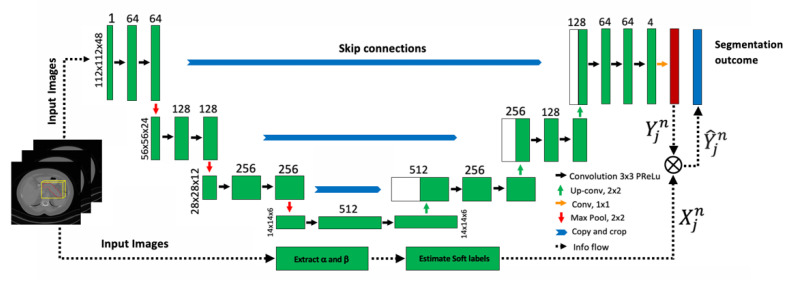
The architectural diagram of the proposed methodology. The probability map for segmentation $Y_{j}^{n}$ obtained from the U-Net is updated to ${\hat{Y}}_{j}^{n}$ by finding the joint probability with soft labels $X_{j}^{n}$ obtained through Bayes probability using α and β.

The standard U-Net architecture was implemented using MONAI 0.7 with the backend of PyTorch 1.8.0 as a network with three down-sampling and three up-sampling steps. The architecture took the 3D volume of the pancreas (cropped image) as the input, with normalized voxel intensities concatenated with the subregional labels as the second channel for training. The loss function was the mean Dice loss generated for each subregion and the focal loss at a 1:1 ratio. The network optimization was realized with an Adam mini-batch gradient descent, whereas the learning rate was 1 × 10^−4^ with a batch size of 2. In each epoch iteration, the probability map $Y_{j}^{n}$, generated by the U-Net model for the *j* training image, is updated to ${\hat{Y}}_{j}^{n}$ by finding the joint probability map as ${\hat{Y}}_{j}^{n}=Y_{j}^{n}*X_{j}^{n}$ before the system performs segmentation. For example, for a pancreas pixel *i*, the normalized probabilities of being part of the head from both models in $X_{j}$ and $Y_{j}$ were multiplied. This new joint probability map ${\hat{Y}}_{j}^{n}$ was then normalized so the sum of all three probabilities for each pixel was exactly 1. The maximum epoch number was set to 500 to obtain the best model performance on the training data, although the algorithm generally converged around 200 epochs and optimized the training *DSC* for three subregions. The training time on 60 training 3D images took around 8 h on an NVIDIA GeForce GTX 2080Ti 10GB GPU. The architectural diagram of the overall methodology is provided in the Figure 2.

### 3.3. Model Testing

The model performed validation on ~20 unique testing scans in each of 4 testing folds. In each testing fold, the Bayes model estimated the probability map $X_{j}^{n}$ for all testing images by using the distribution parameters learned in the *n*th training fold. The U-Net model also estimated $Y_{j}^{n}$ for all testing images, updated to ${\hat{Y}}_{j}^{n}$ by integrating it with $X_{j}^{n}$, and performed segmentation. In addition, the model was also tested on *D*_1_ and *D*_2_ datasets to perform external validation. No training was performed using any image of *D*_1_ and *D*_2_. However, all images of the NIH dataset were grouped as training data to get the best parameters for the model before testing on *D*_1_ and *D*_2_.

## 4. Results and Discussion

**Figure 3:**
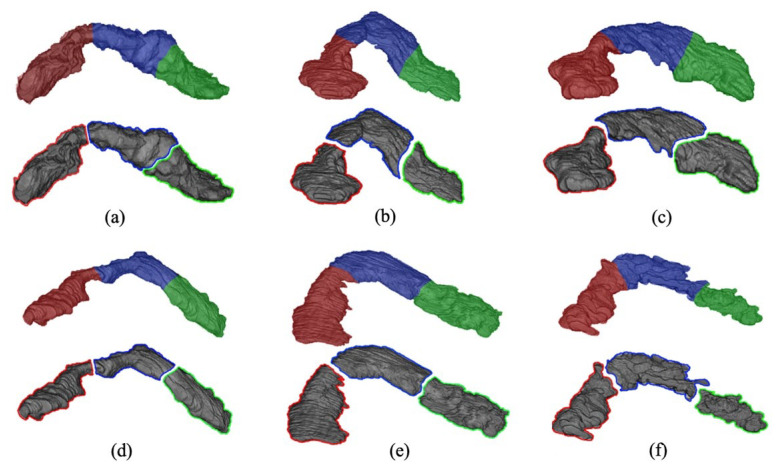
Red (H), Blue (B), Green (T): Best (**a**,**d**), Moderate (**b**,**e**), Worst (**c**,**f**).

The mean overall *DSC* achieved by the proposed segmentation model in the fourfold cross-validation on the NIH dataset was found as 94.5%. Figure 3 shows the outcome of the model with the best, moderate, and low performances. The integration of anatomy-based soft labels into the model improved the overall segmentation *DSC* by 8% on average. This was observed in the segmentation obtained using the U-Net model separately without integration with the Bayes. As the literature does not offer any automated technique for subregional segmentation of the pancreas on CT images, the model performance was assessed by only comparing the predicted segmentation with the benchmark labels.

Testing on *D*_1_ and *D*_2_ resulted in a mean overall *DSC* of 95.6% and 89.9%, respectively. A slightly low *DSC* in the case of *D*_2_ indicates that the U-Net model was challenged by the variation in the texture and morphology of subregions (occurred due to the presence of the tumor or tumor signs). Nevertheless, the performance of the Bayes model was least affected by these changes as the average improvement in the *DSC* on *D*_1_ and *D*_2_ after integration was comparable to those observed on the NIH dataset (i.e., ~8%). This shows reasonable applicability of the anatomy-based soft labels, the replicability of the model to any phase of CT scan, and that it is not limited to portal venous phase only. Table 1 provides the split of the results and insight into how the proposed integration strategy improved the performance of the overall system.

It was observed that the model performance for all three datasets was unaffected by the size variation of the pancreas as the model mostly relies on the percentage proportions of the subregions. Moreover, the overall system during all experiments remained conformed to expectations, as no outlier segmentation was noticed. For example, no single head pixel was incorrectly classified as a tail in the testing images, and vice versa, implying that the approach is stable and robust. Furthermore, failure analysis for all three datasets was performed both quantitatively and qualitatively. Most of the misclassified pixels were found on the border separating body and tail (~67% of overall segmentation failure), typically where most radiologists struggle with delineation. A partial reason for this failure is that there is a relatively less abrupt shape-shift at the body/tail border as compared to the head/body, as shown in Figure 3c. In general, the cases with most segmentation failures have irregular pancreatic morphology, mostly following a U-shape structure.

Furthermore, note that the voxel-size may not always be constant across different CT images. This may raise class-imbalance and affect the performance of the model as the pancreases in different scans would consist of a variable number of slices. However, since our model works on the pre-delineated pancreas, where the number of slices the pancreas is covering in a scan is already known to the model, the impact of variable voxel sizes on the performance is close to none.

Furthermore, the Dice loss is known to be good at handling class imbalance between the foreground and background. For subregional pancreatic segmentation, the actual volume of the pancreas is much smaller than the overall imaging volume, necessitating the use of Dice loss to address the class imbalance problem. Also, the other loss functions, such as the Tversky Loss, can be useful to focus on segmentation in difficult cases. However, since the pancreas’ subregional boundaries do not have large variations across different cases, a less imbalance on the level of training difficulties is expected, which waives the need to apply Tversky Loss. However, it would be an interesting future direction to explore how Tversky Loss performs on regional segmentation of the pancreas.

### Limitation and Future Work

The major limitation of the model is the assumption about the presence of the whole pancreas. The model might get deviated when the pancreas has a history of surgical interventions, such as a Pancreatectomy [27] or Whipple procedure [28] (partial removal of the pancreas). This would also include cases when the size of the subregions varies due to underlying disorders, such as pancreatic inflammation. Note that the segmentation of the whole pancreas is out of the scope of this paper as literature offers several techniques to delineate the whole pancreas in the CT images.

The future work includes extending the model to magnetic resonance (MR) images, which is the second most common imaging modality for PC management after CT [29]. Finally, it would be worth training the model on a larger dataset with high textural variations in the pancreas associated with common pre-conditions, such as pancreatitis and pancreatic cysts.

## 5. Conclusions

This paper presents the first model for the automated 3D segmentation of the delineated pancreas into the head, body, and tail in contrast-enhanced CT images. Using a simple, yet effective, approach, the anatomical constraints of the pancreatic substructures were incorporated in the naïve Bayesian model to generate a probability map that assisted in the prediction of subregional segmentation within the conventional U-Net segmentation model. Three datasets were used in the study; the proposed model was trained on CT scans of the healthy pancreas and tested on CT scans of the healthy, precancerous, and cancerous pancreas. The results are promising but require further training and validation in larger data sets. The accurate segmentation of pancreatic subregions aids reliable analysis and quantification of local morphological changes in the pancreas and can assist in the early diagnosis and treatment planning of PC.

## Figures and Tables

**Table 1:** All values represent percentage *DSC*. ‘Proposed’ refers to the integration of the U-Net with the Bayesian model. Best performance across all datasets is shown in bold.

Data	Model	Head	Body	Tail	Overall
NIH	U-Net	88.8 ± 0.5	86.9 ± 1.4	86.5 ± 1.3	87.5
	Proposed	96.1 ± 1.1	93.8 ± 1.0	92.9 ± 1.1	94.5
*D*_1_	U-Net	86.9 ± 0.6	86.5 ± 0.8	88.5 ± 0.4	87.2
	Proposed	97.0 ± 0.8	95.0 ± 1.2	94.3 ± 0.2	95.6
*D*_2_	U-Net	80.8 ± 1.2	81.3 ± 1.1	82.5 ± 1.0	81.6
	Proposed	89.3 ± 1.5	90.1 ± 0.3	90.6 ± 0.4	89.9

## Data Availability

The NIH data used in this study is publicly available at the NIH data archives. The data labels generated by the authors will be available in their next study or can be requested anytime from the authors.
